# Comparison of cumulative live birth rates between progestin-primed ovarian stimulation protocol and gonadotropin-releasing hormone antagonist protocol in different populations

**DOI:** 10.3389/fendo.2023.1117513

**Published:** 2023-04-18

**Authors:** Ruiqiong Zhou, Mei Dong, Li Huang, Songlu Wang, Lin Fan, Xiangping Liang, Xiqian Zhang, Fenghua Liu

**Affiliations:** Center for Reproductive Medicine, Guangdong Women and Children Hospital, Guangzhou, Guangdong, China

**Keywords:** progestin-primed ovarian stimulation, GnRH antagonist, cumulative live birth rate, preimplantation genetic testing, normal ovarian responder, PCOS, poor ovarian responder

## Abstract

**Objective:**

To compare cumulative live birth rate (LBR) between progestin-primed ovarian stimulation (PPOS) and GnRH antagonist protocols of preimplantation genetic testing (PGT) cycles in different populations.

**Methods:**

This was a retrospective cohort study. A total of 865 patients were enrolled and separate analyses were performed for three populations: 498 patients with predicted normal ovarian response (NOR), 285 patients with PCOS, and 82 patients with predicted poor ovarian response (POR). The primary outcome was cumulative LBR for one oocyte retrieval cycle. The results of response to ovarian stimulation were also investigated, including numbers of oocytes retrieved, MII oocytes, 2PN, blastocysts, good-quality blastocysts, and usable blastocysts after biopsy, as well as rates of oocyte yield, blastocyst formation, good-quality blastocysts, and moderate or severe OHSS. Univariable and multivariable logistic regression analyses were used to identify potential confounders that may be independently associated with cumulative live birth.

**Results:**

In NOR, the cumulative LBR of PPOS protocol was significantly lower than that of GnRH antagonists (28.4% vs. 40.7%; *P*=0.004). In multivariable analysis, the PPOS protocol was negatively associated with cumulative LBR (adjusted OR=0.556; 95% CI, 0.377-0.822) compared to GnRH antagonists after adjusting for potential confounders. The number and ratio of good-quality blastocysts were significantly reduced in PPOS protocol compared to GnRH antagonists (2.82 ± 2.83 vs. 3.20 ± 2.79; *P*=0.032 and 63.9% vs. 68.5%; *P*=0.021), while numbers of oocytes, MII oocytes and 2PN did not show any significant difference between GnRH antagonist and PPOS protocols. PCOS patients had similar outcomes as NOR. The cumulative LBR of PPOS group appeared to be lower than that of GnRH antagonists (37.4% vs. 46.1%; *P*=0.151), but not significantly. Meanwhile, the proportion of good-quality blastocysts in PPOS protocol was also lower compared to GnRH antagonists (63.5% vs. 68.9%; *P*=0.014). In patients with POR, the cumulative LBR of PPOS protocol was comparable to that of GnRH antagonists (19.2% vs. 16.7%; *P*=0.772). There was no statistical difference in the number and rate of good-quality blastocysts between the two protocols in POR, while the proportion of good-quality blastocysts appeared to be higher in PPOS group compared to GnRH antagonists (66.7% vs. 56.3%; *P*=0.182). In addition, the number of usable blastocysts after biopsy was comparable between the two protocols in three populations.

**Conclusion:**

The cumulative LBR of PPOS protocol in PGT cycles is lower than that of GnRH antagonists in NOR. In patients with PCOS, the cumulative LBR of PPOS protocol appears to be lower than that of GnRH antagonists, albeit lacking statistical difference, whereas in patients with diminished ovarian reserve, the two protocols were comparable. Our findings suggest the need for caution when choosing PPOS protocol to achieve live births, especially for normal and high ovarian responders.

## Introduction

Controlled ovarian stimulation (COS) is a key step in assisted reproductive technology (ART) (1). In addition to the use of gonadotropins to recruit multiple follicles, it is mandatory to use a drug to prevent luteinizing hormone (LH) surge and premature ovulation. Gonadotropin-releasing hormone (GnRH) antagonist protocol has emerged as a routine ovulation stimulation protocol in recent decades due to its comparable convenience, safety, and efficacy compared to GnRH agonists (2–4). However, GnRH antagonists are expensive and sometimes difficult to manage with high rates of cycle cancellation and premature LH surges (5–7). Progesterone and its derivatives have recently emerged as alternatives to inhibit premature ovulation during ovarian stimulation, while their efficacy and safety remain to be further investigated. Numerous studies have demonstrated that progesterone has a significant inhibitory effect on LH levels during ovarian stimulation in the luteal phase (8, 9). Letterie et al. demonstrated that a combination of ethinyl estradiol and norethindrone from day 6 or 8 of the menstrual cycle allowed follicular development, while inhibiting the mid-cycle LH surge during COS (10). In 2015, Kuang et al. proposed a new ovarian stimulation protocol, called progestin-primed ovarian stimulation (PPOS), confirming that Medroxyprogesterone 17-acetate (MPA) can prevent premature LH surges during follicular phase ovarian stimulation (11).

In past decades, when IVF relied on fresh embryo transfer, progesterone could not be considered during COS as early exposure to progesterone could lead to embryo-endometrium asynchrony (9). Advances in vitrification have made cryopreservation and thawing of embryos in a reliable manner, which has eliminated concerns about the deleterious effects of progesterone exposure on endometrial receptivity (12). Therefore, PPOS protocol may be a suitable option when fresh embryo transfer is not required, such as in oocyte donation, fertility preservation and preimplantation genetic testing (PGT) cycles (13).

Although delaying embryo transfer can avoid the adverse effects of early endometrial exposure to progesterone, many studies have reported concerns about prolonged exposure to progesterone during follicular development. It was shown that elevated progesterone was significantly associated with adverse effects on embryo quality and cumulative live birth rate (LBR) (14–16). Previous studies have shown that progestins were as effective as GnRH antagonists in preventing LH surges during COS. Most studies have found that the response to ovarian stimulation, e.g., oocyte retrieved, was similar in PPOS protocol and GnRH antagonists. However, reports on pregnancy outcomes were inconclusive when comparing PPOS protocol with GnRH antagonists (17–24). The cumulative LBR is considered the most important patient-centered outcome for assessing the success of ART cycles (25), which has rarely been mentioned in previous studies. To our knowledge, only one published study investigated the cumulative LBR of GnRH antagonists and PPOS protocol in those with poor ovarian response and found similar cumulative LBR for PPOS protocol and GnRH antagonists (24). However, their study did not take into account the possible bias of including fresh embryo transfer in GnRH antagonist protocol, and there were no data on embryo quality. PGT cycle may be an appropriate model for comparing pregnancy outcomes between PPOS protocol and GnRH antagonists.

The purpose of this study is to compare the cumulative LBR of PPOS protocol and GnRH antagonists in PGT cycles, with a particular focus on different populations.

## Methods

### Study design and participants

This study was a retrospective study performed at the Reproductive Medicine Centre of Guangdong Women and Children’s Hospital. The hospital’s Institutional Review Board for Ethics has approved the study protocol. The study included patients who underwent PGT using GnRH antagonist protocol or PPOS protocol between January 2017 and August 2020. The inclusion criteria were as follows: (i) maternal age ≤ 40 years; (ii) PGT cycles. The exclusion criteria were as follows: (i) uterine abnormalities and intrauterine adhesion; (ii) cancelled embryo biopsy and repeated cycles; (iii) cycles using donated oocytes or sperm; (iv) core data missing.

In this study, three populations were analysed separately: predicted normal ovarian responders (NOR) with normal ovarian reserve and regular menstrual cycle (defined as a cycle length of 21–35 days), patients with PCOS and predicted poor ovarian responders (POR). PCOS was diagnosed according to the Rotterdam ESHRE/ASRM-Sponsored PCOS consensus workshop group (26). POR was considered when antral follicle count (AFC) < 5 or AMH < 1.1 ng/ml or previous adverse ovarian response (27).

### Ovarian stimulation

**Figure 1:**
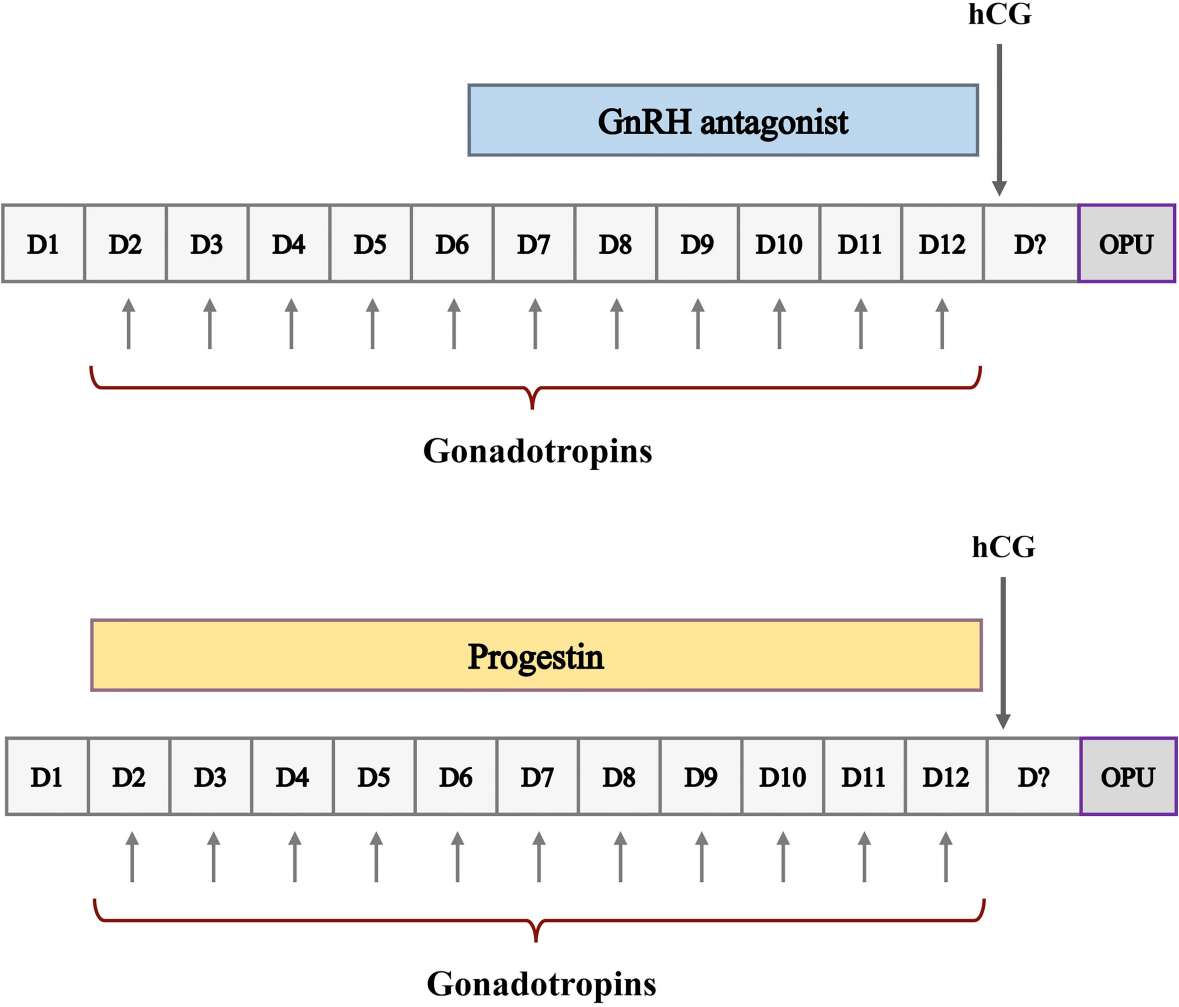
Schematic representation of ovarian stimulation protocols for ART. D, cycle day; CD21, cycle day 21; OPU, oocyte pick-up.

A simplified schematic of ovarian stimulation using the GnRH antagonist protocol and the PPOS protocol is shown in **Figure 1**. The GnRH antagonist and PPOS protocols are the two commonly used ovarian stimulation protocols for PGT cycles in our center. For the GnRH antagonist protocol, recombinant follicle-stimulating hormone (rFSH) (Gnoal-f; Merck Serono or Puregon; MSD, Organon) at a dose of 100 to 300 IU per day was administered on day 2 or 3 of the menstrual cycle. GnRH antagonists (Ganirelix; MSD, Organon) were started flexibly when at least one follicle was ≥ 12 mm at a daily dose of 0.25 mg and continued until the day of hCG trigger. For the PPOS protocol, a daily dose of 10 mg medroxyprogesterone acetate (MPA) in combination with 100 to 300 IU of rFSH was administered on day 2 or 3 of the menstrual cycle until hCG trigger day. The ovarian response was assessed by ultrasound and serum hormone levels, which were monitored form the first day of stimulation and measured every 3 to 4 days until the trigger day. In both protocols, dose adjustment was based on ovarian response assessed by ultrasound and serum hormone levels. When at least three follicles measured 17 mm or at least two follicles reached 18 mm in diameter, hCG was administered at a dose of 6000 to 10000 IU to induce oocyte maturation. Oocyte retrieval was performed 35-36 hours later by transvaginal ultrasound-guided follicular aspiration, and only oocytes with the first polar body released (metaphase II) were fertilized by intracytoplasmic sperm injection (ICSI) immediately after the denudation procedure.

### Blastocyst grading and preimplantation genetic testing

All embryos were cultured to the blastocyst stage on day 5 or 6. According to the Gardner criteria (28), blastocyst morphology score was based on three components: blastocyst expansion, inner cell mass, and trophectoderm development. Two embryologists independently assessed blastocyst morphology prior to trophectoderm biopsy. If blastocyst grade ≥ 3 BC, we performed biopsy followed by vitrification. Blastocysts with both a ‘C’ type inner cell mass and a ‘C’ type trophectoderm were discarded. Good-quality blastocysts were defined as ≥ 3 BB (AA, AB, BA and BB). On the day of biopsy, 5-8 trophectoderm cells were gently aspirated into the biopsy pipette followed by a laser-assisted removal from the rest of blastocyst. PGT was mainly used in patients with advanced maternal age (≥ 38y), recurrent spontaneous abortion (RSA) and repeated implantation failure, couples with monogenic disease, and couples with chromosomal structural rearrangements.

### Frozen-thawed embryo transfer

After biopsy, only one available blastocyst was transferred at a time, with preference given to higher quality blastocysts based on morphological criteria. Embryos were thawed on the morning of the transfer day, and post-thaw embryos with ≥ 50% blastomeres intact were considered viable. Detailed vitrification and thawing protocols have been reported in our previous study (29). In our reproductive center, we mainly applied three endometrial preparation protocols based on patient characteristics, preference and/or at the discretion of physician, as no regimen was superior to another for endometrial preparation in terms of clinical outcomes (30–32). The method of luteal phase support depends on patient’s preference, as there is no clear medical evidence that using one option is better than another (33–35). Intramuscular progesterone (40 mg once daily) or a combination of vaginal progesterone sustained-release gel (Crinone 8%, 90mg once daily) and oral progesterone (Dydrogesterone, 10 mg twice daily) was administered until 10 weeks of gestation.

### Outcome measures

The primary outcome was the cumulative live birth rate for one oocyte retrieval cycle. The follow-up period was 2 years or until live birth occurred. The cumulative LBR was calculated by dividing the number of patients with live births at 24 weeks of gestation or more by the total number of patients assigned to the group. The time to live birth was the time from cycle start to live birth within an ART cycle. The outcomes of response to ovarian stimulation included numbers of oocytes retrieved, metaphase II oocytes (MII), 2 pronuclear fertilized oocytes (2PN), blastocysts, good-quality blastocysts, and usable blastocysts after biopsy, as well as rate of oocyte yield (the ratio between the total number of oocytes retrieved and the number of follicles with a mean diameter > 10 mm on the day of trigger), rate of blastocyst formation (the ratio between the number of blastocysts formed and the number of 2PN cultured), rate of good-quality blastocysts (the ratio between the number of good-quality blastocysts and the number of blastocysts formed), and rate moderate or severe OHSS (36). A usable blastocyst after biopsy was defined as an euploid embryo for PGT-A, an euploid embryo without pathogenic phenotype for PGT-M, and a balanced embryo for PGT-SR.

### Statistical analysis

Statistical analyses were performed using the SPSS statistical package (SPSS, version 22.0). *P-*value < 0.05 was considered statistically significant. The Kolmogorov–Smirnov test was used to determine whether continuous variables were normally distributed. Continuous variables were presented as mean with standard deviation (mean ± SD). Student’s t-test or Mann-Whitney U-test was conducted to assess significant differences where appropriate. Categorical variables were described as number with percentage and compared by Pearson’s chi-square test or Fisher’s exact test.

We performed separate analyses on three populations: NOR, PCOS patients, and POR. Univariable and multivariable logistic regression analyses were used to identify potential confounders that may be independently associated with cumulative live birth. Confounders were assessed by univariable analyses (**Supplementary Table 1**) and then added to the multivariable regression models for adjustment. Variables with significance at *P* < 0.10 or more in the univariable analysis and those that may potentially have an effect on cumulative LBR (e.g., AFC) were included in the multivariable model. The following confounders were entered in multivariable models: age, BMI, AFC, days of stimulation, estradiol (E_2_) on hCG trigger day, PGT method, and RSA. Crude and adjusted odds ratio (OR) and 95% confidence interval (CI) were calculated for the cumulative LBR in the PPOS group compared to the GnRH antagonist group.

## Results

### Patient characteristics

**Figure 2:**
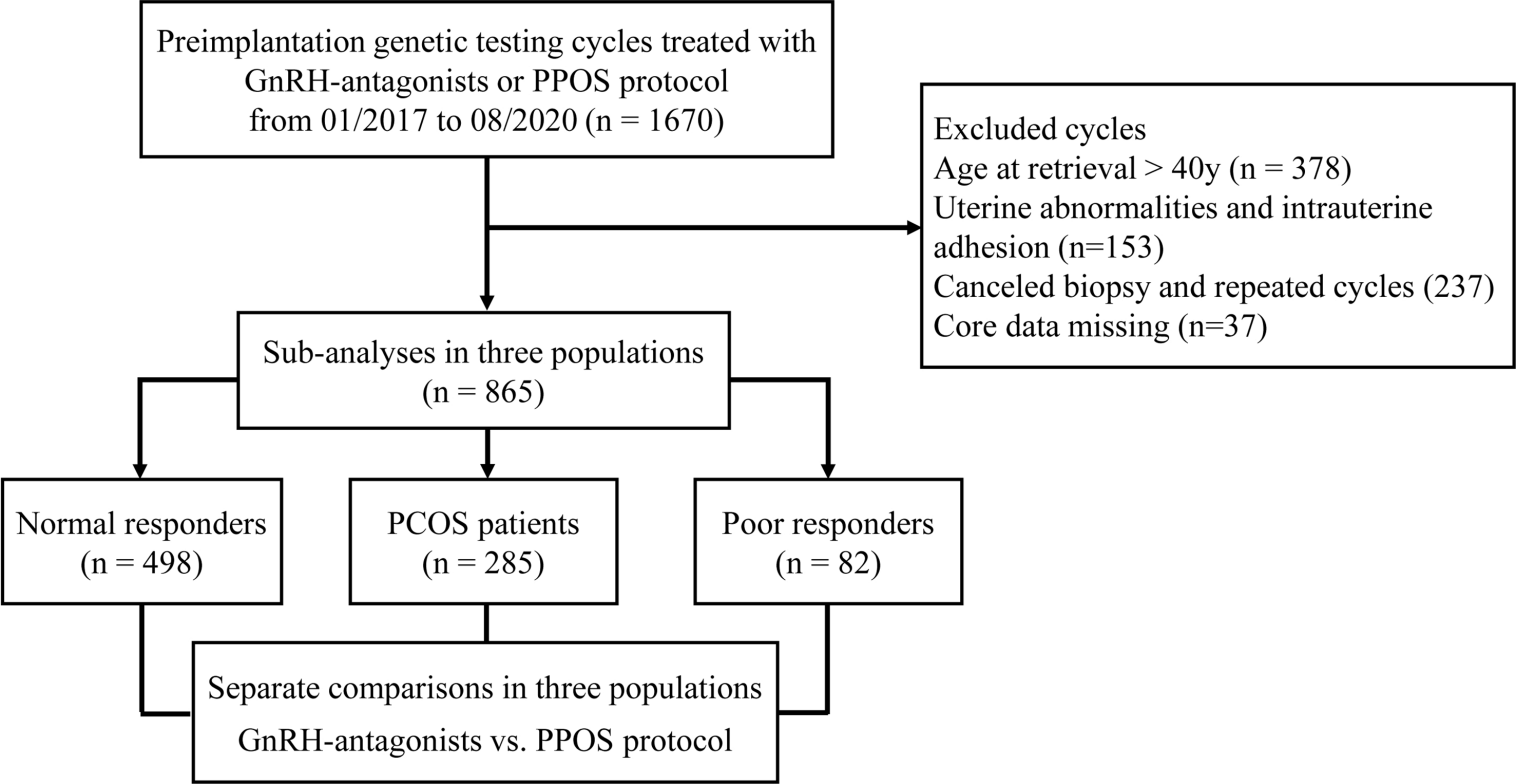
Flow chart of the study.

The flowchart of the study is presented in **Figure 2**. A total of 865 patients who met the inclusion and exclusion criteria were included in this study. Separate comparisons were made in three populations: 498 patients with NOR, 285 patients with PCOS, and 82 patients with POR. The baseline characteristics of GnRH antagonist and PPOS protocols were compared in each of the three populations separately, and they did not show any significant differences between two groups (**Table 1**).

**Table 1:** Baseline characteristics of patients according to ovarian stimulation protocols in three populations.

Parameters	Normal responders			PCOS patients			Poor responders		
	GnRH-antagonist (n=248)	PPOS (n=250)	*P* value	GnRH-antagonist (n=178)	PPOS (n=107)	*P* value	GnRH-antagonist (n=30)	PPOS (n=52)	*P* value
Age (years)	32.06 ± 4.15	32.65 ± 4.74	0.156	29.99 ± 3.89	29.59 ± 3.40	0.554	35.50 ± 4.27	35.63 ± 4.05	0.888
BMI (kg/m^2^)	22.05 ± 2.91	21.94 ± 2.60	0.893	21.72 ± 2.89	21.51 ± 2.72	0.576	21.51 ± 2.87	22.29 ± 3.25	0.264
AFC	12.70 ± 4.95	12.20 ± 4.69	0.276	23.21 ± 5.60	24.04 ± 7.35	0.720	5.80 ± 3.53	5.62 ± 2.74	0.756
AMH (ng/ml)	3.90 ± 2.23	3.76 ± 2.19	0.499	10.03 ± 4.65	9.68 ± 4.57	0.524	0.79 ± 0.44	0.86 ± 0.37	0.707
Duration of infertility (years)	2.11 ± 2.57	2.21 ± 2.91	0.306	2.18 ± 2.07	1.97 ± 1.92	0.473	2.05 ± 2.49	3.05 ± 3.67	0.299
Type of infertility			0.167			0.409			0.173
Primary	46 (18.5)	59 (23.6)		53 (29.8)	27 (25.2)		2 (6.7)	9 (17.3)	
Secondary	202 (81.5)	191 (76.4)		125 (70.2)	80 (74.8)		28 (93.3)	43 (82.7)	
PGT treatment			0.550			0.815			0.427
PGT-A	105 (42.3)	116 (46.4)		49 (27.5)	33 (30.8)		23 (76.7)	35 (67.3)	
PGT-M	37 (14.9)	39 (15.6)		33 (18.5)	20 (18.7)		2 (6.7)	2 (3.8)	
PGT-SR	106 (42.7)	95 (38.0)		96 (53.9)	54 (50.5)		5 (16.7)	15 (28.8)	
Recurrent spontaneous abortion	96 (38.7)	89 (35.6)	0.473	61 (34.3)	33 (30.8)	0.551	14 (46.7)	26 (50.0)	0.771
**PGT cycle rank**			0.566			0.641			0.503
**First**	190 (76.6)	186 (74.4)		158 (88.8)	93 (86.9)		19 (63.3)	29 (55.8)	
**High order**	58 (23.4)	64 (25.6)		20 (11.2)	14 (13.1)		11 (36.7)	23 (44.2)	
**Presence of tubal factor**	63 (25.4)	58 (23.2)	0.567	49 (27.5)	24 (22.4)	0.340	8 (26.7)	16 (30.8)	0.694
**Presence of endometriosis**	13 (5.2)	10 (4.0)	0.509	4 (2.2)	2 (1.9)	0.830	2 (6.7)	5 (9.6)	0.645
**Presence of male factor**	21 (8.5)	16 (6.4)	0.379	8 (4.5)	6 (5.6)	0.674	0	1 (1.9)	0.445

Continuous variables are presented as mean ± SD and Mann-Whitney U-test was conducted to assess significant differences; Categorical variables are presented as number (percentage) and Pearson’s chi-square test was conducted to compare differences. PCOS, polycystic ovary syndrome; PPOS, progestin-primed ovarian stimulation; BMI, body mass index; AFC, antral follicle count; AMH, anti-mullerian hormone; PGT-A, preimplantation genetic testing for aneuploidy; M, monogenic disorder; SR, chromosomal structural rearrangement.

### Predicted normal ovarian responders

In NOR, there were no significant differences between GnRH-antagonist and PPOS groups in terms of gonadotropin dose and E2 on hCG trigger day (**Table 2**). Nevertheless, the duration of stimulation was longer in the GnRH antagonist group than in the PPOS group (9.99 ± 1.35 vs. 9.63 ± 1.37; *P*=0.006). There were no significant differences in numbers of oocytes retrieved, MII and 2PN between GnRH antagonist and PPOS groups. Notably, oocyte yield was significantly higher in the GnRH antagonist group than in the PPOS group (96.5% vs. 93.7%; *P*<0.001). The number and rate of blastocysts did not show any significant difference between GnRH antagonist and PPOS groups, yet the number and ratio of good-quality blastocysts were significantly reduced in the PPOS protocol compared to GnRH antagonists (2.82 ± 2.83 vs. 3.20 ± 2.79; *P*=0.032 and 63.9% vs. 68.5%; *P*=0.021). After biopsy, the number of available blastocysts was comparable between the two groups. In addition, there was no significant difference in the incidence of moderate or severe OHSS between the two groups.

**Table 2:** Response to ovarian stimulation and cumulative live birth in three populations.

Parameters	Normal responders			PCOS patients			Poor responders		
	GnRH-antagonist(n=248)	PPOS (n=250)	*P* value	GnRH-antagonist(n=178)	PPOS(n=107)	*P* value	GnRH-antagonist(n=30)	PPOS(n=52)	*P* value
Gonadotropin dose (IU)	2183 ± 677	2199 ± 648	0.680	1433 ± 486	1406 ± 446	0.805	2380 ± 866	2545 ± 767	0.603
Days of stimulation	9.99 ± 1.35	9.63 ± 1.37	0.006	10.05 ± 1.75	9.58 ± 1.38	0.022	8.63 ± 2.46	8.98 ± 1.79	0.910
Estradiol on hCG trigger day (pg/ml)	3328 ± 1658	3464 ± 1721	0.324	4759 ± 2179	5479 ± 2780	0.106	1760 ± 1084	1624 ± 1074	0.583
No. of oocytes retrieved	16.35 ± 7.61	15.67 ± 7.00	0.571	24.08 ± 10.82	24.20 ± 10.56	0.972	6.93 ± 4.19	6.50 ± 4.61	0.439
Oocyte yield	4054/4202 (96.5)	3918/4181 (93.7)	< 0.001	4286/4510 (95.0)	2589/2841 (91.1)	< 0.001	208/217 (95.9)	338/348 (97.1)	0.414
No. of MII	13.03 ± 6.26	12.73 ± 6.15	0.770	18.66 ± 7.94	19.44 ± 8.25	0.580	5.53 ± 3.59	5.23 ± 3.83	0.735
No. of 2PN	10.07 ± 5.17	9.75 ± 4.74	0.678	14.21 ± 5.68	14.46 ± 6.14	0.983	4.37 ± 3.48	3.88 ± 3.17	0.655
No. of blastocysts	4.67 ± 3.18	4.41 ± 3.32	0.175	6.94 ± 3.62	6.81 ± 4.03	0.535	2.13 ± 2.30	1.85 ± 2.05	0.565
Rate of blastocyst formation	1159/2439 (47.5)	1103/2387 (46.2)	0.362	1236/2490 (49.6)	729/1495 (48.8)	0.592	64/132 (48.5)	96/198 (48.5)	1.000
No. of good-quality blastocysts	3.20 ± 2.79	2.82 ± 2.83	0.032	4.79 ± 3.39	4.33 ± 3.29	0.237	1.20 ± 1.83	1.23 ± 1.55	0.831
Rate of good-quality blastocysts	794/1159 (68.5)	705/1103 (63.9)	0.021	852/1236 (68.9)	463/729 (63.5)	0.014	36/64 (56.3)	64/96 (66.7)	0.182
No. of available blastocysts after biopsy	1.97 ± 1.81	1.76 ± 1.79	0.145	2.77 ± 2.28	2.66 ± 2.23	0.691	0.83 ± 1.02	0.75 ± 1.19	0.369
Moderate or severe OHSS rate	2 (0.8)	4 (1.6)	0.417	7 (3.9)	10 (9.3)	0.062	0	0	/
Cumulative live birth rate	101 (40.7)	71 (28.4)	0.004	82 (46.1)	40 (37.4)	0.151	5 (16.7)	10 (19.2)	0.772
Time to live birth (months)	13.34 ± 2.82	14.36 ± 3.53	0.055	14.04 ± 4.36	14.51 ± 3.63	0.075	13.00 ± 1.05	12.96 ± 1.79	0.806
**No. of embryo transfers that reach live birth or end of observation**	1.02 ± 0.87	0.95 ± 0.80	0.389	1.16 ± 0.90	1.17 ± 1.09	0.613	0.63 ± 0.72	0.58 ± 0.70	0.724

Continuous variables are presented as mean ± SD and Mann-Whitney U-test was conducted to assess significant differences; Categorical variables are presented as number (percentage) and

Pearson’s chi-square test was conducted to compare differences. PCOS, polycystic ovary syndrome; PPOS, progestin-primed ovarian stimulation; MII, mature oocytes; 2PN, 2 pronuclear; OHSS, ovarian hyperstimulation syndrome. Oocyte yield was defined as the ratio between the total number of oocytes retrieved and the number of follicles with a mean diameter > 10 mm on the day of trigger.

The cumulative LBR was significantly lower in the PPOS group than in the GnRH antagonist group (28.4% vs. 40.7%; *P*=0.004) (**Table 2**). There was no significant difference in mean time to live birth between the two groups, while there was a trend towards longer time in the PPOS group compared to the GnRH antagonist group (14.36 ± 3.53 vs. 13.34 ± 2.82; *P*=0.055). In multivariable analysis, the PPOS protocol was negatively associated with cumulative LBR (adjusted OR=0.556; 95% CI, 0.377-0.822) compared to GnRH antagonists after adjusting for age, BMI, AFC, days of stimulation, E2 on hCG trigger day, PGT method, and RSA (**Table 3**). BMI was also an independent predictor of cumulative LBR in the multivariable model (adjusted OR=0.916; 95% CI, 0.851-0.986).

**Table 3:** Multivariable regression analysis of cumulative live birth in different populations.

Parameters	Normal responders Adjusted OR (95% CI)	PCOS Adjusted OR (95% CI)	Poor responders Adjusted OR (95% CI)
Age	0.975 (0.927-1.025)	0.984 (0.917-1.056)	0.909 (0.760-1.086)
BMI	0.916 (0.851-0.986)	0.981 (0.897-1.073)	0.838 (0.658-1.069)
AFC	1.005 (0.963-1.049)	0.952 (0.911-0.994)	1.050 (0.837-1.317)
Days of stimulation	1.062 (0.915-1.233)	1.094 (0.941-1.273)	1.234 (0.748-2.037)
Estradiol on hCG trigger day	0.999 (0.999-1.000)	0.999 (0.999-1.000)	1.001 (1.000-1.002)
PGT method			
PGT-A	Reference	Reference	Reference
PGT-M	1.522 (0.748-3.098)	1.129 (0.470-2.714)	0.138 (0.006-3.383)
PGT-SR	1.111 (0.634-1.947)	1.197 (0.604-2.372)	0.344 (0.049-2.433)
Recurrent spontaneous abortion (Yes vs. No)	1.214 (0.717-2.056)	1.273 (0.678-2.392)	1.885 (0.378-9.401)
PPOS vs. GnRH-antagonist	0.556 (0.377-0.822)	0.767 (0.460-1.279)	2.126 (0.454-9.965)

OR, odds ratio; CI, confidence interval; PCOS, polycystic ovary syndrome; BMI, body mass index; AFC, antral follicle count; AMH, anti-mullerian hormone; PGT-A, preimplantation genetic testing for aneuploidy; M, monogenic disorder; SR, chromosomal structural rearrangement; PPOS, progestin-primed ovarian stimulation.

### Women with PCOS

In patients with PCOS, there were no significant differences between the GnRH antagonist and PPOS groups in regards to gonadotropin dose, E2 on hCG trigger day, and numbers of oocytes retrieved, MII, 2PN and blastocysts, whereas the stimulation time (10.05 ± 1.75 vs. 9.58 ± 1.38; *P*=0.022) and oocyte yield (95.0% vs. 91.1%; *P*<0.001) were higher in the GnRH antagonist group than in the PPOS group, with results similar to those of NOR (**Table 2**). The rate of good-quality blastocysts was significantly lower with the PPOS protocol compared to GnRH antagonists (63.5% vs. 68.9%; *P*=0.014), but there was no significant difference in the average number of good-quality blastocysts. Likewise, the number of blastocysts available after biopsy was comparable between the two groups. Additionally, the incidence of moderate or severe OHSS tended to be higher in the PPOS group than in the GnRH antagonist group, although not statistically significant.

In PCOS patients, the cumulative LBR tended to be lower in the PPOS group compared to the GnRH antagonist group, although not statistically significant (37.4% vs. 46.1%; *P*=0.151). Similar to the NOR population, there was a trend towards longer time to live birth in the PPOS group compared to the GnRH antagonist group (14.51 ± 3.63 vs. 14.04 ± 4.36; *P*=0.075). After controlling for major covariates, there was no evidence of a significant association between the PPOS protocol and cumulative live birth (adjusted OR = 0.767; 95% CI, 0.460-1.279), while AFC was inversely associated with cumulative LBR in patients with PCOS (adjusted OR = 0.952; 95% CI, 0.911-0.994) (**Table 3**).

### Predicted poor ovarian responders

In patients with POR, the results of response to ovarian stimulation were mostly similar in the GnRH antagonist and PPOS groups (**Table 2**). However, the proportion of good-quality blastocysts was unexpectedly higher in the PPOS protocol compared with GnRH antagonists, although not statistically significant (66.7% vs. 56.3%; *P*=0.182), which may be related to insufficient patient enrolment. In addition, the cumulative LBR was comparable between the two groups (16.7% vs. 19.2%; *P*=0.772). In POR, no evidence of a significant association between the PPOS protocol and cumulative live birth was found in either univariable (OR=1.190; 95%CI, 0.365-3.883) or multivariable analyses (adjusted OR=2.126; 95%CI, 0.454-9.965) (**Supplementary Table 1** and **Table 3**).

## Discussion

Progestins have recently been used as a substitute for GnRH antagonists to prevent premature LH surge due to the well-established application of vitrification technology (13). The PPOS protocol has greater flexibility and new potential to improve clinical care for patients, but its long-term efficacy and safety, including oocyte competence and cumulative LBR, remain to be demonstrated (37).

This study is the first to compare the cumulative LBR of PPOS protocol and GnRH antagonists in different populations. In NOR, the cumulative LBR of PPOS protocol was significantly lower than that of GnRH antagonists. Surprisingly, the number and ratio of good-quality blastocysts were significantly reduced in PPOS protocol compared to GnRH antagonists, while the numbers of oocytes, MII oocytes and 2PN did not show any significant difference between GnRH antagonist and PPOS protocols. Notably, PCOS patients had similar outcomes as NOR. The cumulative LBR of PPOS group appeared to be lower than that of GnRH antagonists, but not significantly, which may be related to insufficient number of patients enrolled. Meanwhile, the proportion of good-quality blastocysts in PPOS protocol was also lower compared to GnRH antagonists. Of interest, the cumulative LBR of PPOS protocol was comparable to that of GnRH antagonists in patients with POR, which was consistent with the results of previous studies (24). In addition, there was no statistical difference in the number and rate of good-quality blastocysts between the two protocols in POR, while the proportion of good-quality blastocysts appeared to be higher in PPOS group compared to GnRH antagonists, but not significantly.

Studies have shown that elevated progesterone during stimulation adversely affects the formation of good-quality embryos and cumulative live births (14–16). Previously, progestins were compared with GnRH antagonists in 11 studies (17–24, 38–41). Most studies have shown that the response to ovarian stimulation, including gonadotropin consumption, and numbers of oocytes and MII oocytes, was similar between PPOS protocol and GnRH antagonists (37), which is consistent with our findings. Nevertheless, previous studies on pregnancy outcomes have been inconclusive. In a randomized clinical study, the PPOS protocol had unexpectedly significantly lower clinical pregnancy (29.9% versus 42.5%) and live birth rates (27.4% versus 36.9%) compared to GnRH antagonists. As for their primary outcome, the number of MII oocytes in oocyte donation cycles was similar in the two groups (17). In contrast, a meta-analysis of previous randomized controlled trials showed no difference in clinical pregnancy rates and live birth or ongoing pregnancy rates with the PPOS protocol compared to other protocols, yet they did not include studies on cumulative LBR and it may be better to compare the PPOS protocol separately with several other protocols (41). A retrospective study showed similar implantation, clinical pregnancy and live birth rates in patients with oocyte donation using PPOS protocol and GnRH antagonists (23). However, their study reported a significant increase in the number of oocytes retrieved in the PPOS group, and it is possible that this group have included oocyte donors with higher ovarian reserve. Most studies have focused on pregnancy outcomes per embryo transfer rather than cumulative LBR per ovarian stimulation, which may be the most meaningful outcome for infertile patients. Only one published study compared the cumulative LBR of GnRH antagonists and PPOS protocol in patients with poor ovarian response and found no significant difference between the two protocols (24). However, their study did not account for the bias arising from the inclusion of fresh embryo transfer in GnRH antagonist protocol, and there were no data on embryo quality.

Previous studies have been conducted mainly in oocyte donation cycles or non-freeze-all cycles using autologous oocytes. In oocyte donation cycles, patient characteristics may vary considerably between donors and recipients, which may increase the potential for outcome bias when comparing different stimulation protocols. In non-freeze-all cycles, fresh embryo transfer may be the preferred choice for GnRH antagonists when conditions permit, whereas PPOS protocol adopts a freeze-all strategy. Therefore, there is a need to explore PPOS protocol in cycles that do not require fresh embryo transfer. The PGT cycle may be a more appropriate model for comparing these results to reduce bias.

Progestins have limited data on embryo quality compared to GnRH antagonists, especially blastocyst quality and euploid blastocysts. A recent case-control study found similar numbers of euploid blastocysts in patients undergoing PGT cycles with PPOS and GnRH antagonist protocols (42). However, it did not investigate morphological blastocyst quality and pregnancy outcomes. Our results are consistent with their finding that the number of blastocysts available after biopsy was comparable between the two protocols in all three populations. In addition, our study investigated blastocyst morphology scores for the first time and found that the percentage of good-quality blastocysts was significantly lower in the PPOS protocol than in the GnRH antagonists in both NOR and PCOS patients, while in POR population, the rate of good-quality blastocysts appeared to be higher in the PPOS protocol. Studies have shown that, in addition to euploid status, blastocyst quality based on morphology score is a factor that is highly correlated with implantation potential and pregnancy outcomes (28, 43–47). Given that blastocyst score is a predictor of live birth, a reduction in high-quality blastocysts in PPOS cycles compared to GnRH antagonists may be associated with a lower cumulative LBR. More studies are needed to confirm whether blastocyst quality and euploid blastocysts differ between patients treated with PPOS protocol and GnRH antagonists.

In our study, the main focus was on cumulative LBR, which could provide better data to assess the effectiveness and safety of ovarian stimulation methods (25). Earlier research has shown that the cumulative LBR increases with the number of oocytes obtained (48). However, in our study, although similar numbers of oocytes, MII oocytes and 2PN were obtained with GnRH antagonists and PPOS protocol, the cumulative LBR and good-quality blastocyst rate were significantly lower with the PPOS protocol in patients with NOR and PCOS. Moreover, in patients with NOR and PCOS, oocyte yield was significantly lower in the PPOS group than in GnRH antagonists, although ovarian reserve was similar in the two protocols in each population. However, it is unknown whether the use of progesterone during stimulation affects oocyte maturation and the underlying mechanism.

Notably, previous studies comparing PPOS protocol with GnRH antagonists have been conducted mainly in oocyte donors, diminished ovarian reserve and undefined populations, and rarely in NOR and PCOS patients. The response to ovarian stimulation is limited by ovarian reserve, and the PPOS protocol should be studied in different populations to see if it actually produces as good a response as GnRH antagonists. Additionally, many previous studies used human menopausal gonadotropin (hMG) to induce ovarian stimulation, whereas our study used rFSH as the gonadotropin, which may have influenced the response to COS to some extent (11, 49).

This study has some limitations due to its retrospective nature. First, although great efforts have been made to reduce sources of bias, particularly by using multivariable regression models and to differentiate homogenous populations, however, patients were assigned to different protocols in a non-randomized manner. Second, cost-effectiveness was not analyzed in this study because our electronic health care system does not show patient costs. Third, our study included cycles of different PGT methods, which could be a potential confounder affecting pregnancy outcomes. However, it was observed that the various PGT methods of the two protocols did not differ in the three populations, and PGT methods were not associated with cumulative LBR in our multivariable models. Fourth, our study did not compare the use of different progestins in PPOS protocol, as our center only use MPA for PPOS regimen. Finally, we did not analyze the obstetrical and neonatal outcomes of the PPOS protocol because only a small sample of live birth was available in this study, and further studies are needed to confirm the perinatal outcomes and long-term safety of offspring due to its short duration of use and limited amount of data (37, 50). Given that cumulative LBR is a complex multifactorial outcome that cannot be explained by ovarian stimulation protocols alone, it should be interpreted with caution.

In conclusion, the cumulative LBR of PPOS protocol in PGT cycles is lower than that of GnRH antagonists in normal ovarian responders. In patients with PCOS, the cumulative LBR of PPOS protocol appears to be lower than that of GnRH antagonists, albeit lacking statistical difference, whereas in patients with diminished ovarian reserve, the two protocols were comparable. Our findings suggest the need for caution when choosing PPOS protocol to achieve live births, especially for normal and high ovarian responders. To confirm our findings, more well-designed studies are needed to elucidate the effects of PPOS protocol on oocyte development, cumulative LBR and neonatal outcomes, and further studies are needed to explore the underlying mechanism.

## Data availability statement

The raw data supporting the conclusions of this article will be made available by the authors, without undue reservation.

## Author contributions

RZ and FL conceived and designed the study. All the authors analyzed and interpreted the data.LH contributed to the data collection. RZ performed the statistical analysis and wrote the article. FL and XZ revised the article. All authors contributed to the article and approved the submitted version.
