# Supplementary material for: Comparison of cumulative live birth rates between progestin-primed ovarian stimulation protocol and gonadotropin-releasing hormone antagonist protocol in different populations
Source: Front Endocrinol (Lausanne). 2023 Apr 18;14:1117513. doi: 10.3389/fendo.2023.1117513 (PMC10151746; doi:10.3389/fendo.2023.1117513)
Supplement: Supplementary file 1 [file Table_1.docx]

**Supplementary Table 1** Univariable regression analysis of cumulative live birth in different populations.

| **Parameters** | **Normal responders**  **Crude OR (95%CI)** | ***P* value** | **PCOS**  **Crude OR (95%CI)** | ***P* value** | **Poor responders**  **Crude OR (95%CI)** | ***P* value** |
| --- | --- | --- | --- | --- | --- | --- |
| Age | 0.956 (0.917-0.997) | 0.035 | 1.002 (0.940-1.067) | 0.956 | 0.865 (0.760-0.985) | 0.028 |
| BMI  AFC  AMH  Duration of infertility  Secondary vs. primary infertility  Gonadotropin dose  Days of stimulation  Hormones on hCG trigger day  Estradiol  LH  Progesterone  PGT treatment  PGT-A  PGT-M  PGT-SR  RSA (Yes vs. No)  PPOS vs. GnRH-antagonist | 0.921 (0.859-0.988)  1.012 (0.974-1.051)  1.032 (0.951-1.121)  0.985 (0.920-1.055)  1.336 (0.837-2.131)  0.999 (0.999-1.000)  1.066 (0.931-1.221)  1.000 (0.999-1.000)  0.987 (0.882-1.104)  0.954 (0.809-1.124)  Reference  1.602 (0.936-2.741)  1.203 (0.802-1.805)  1.004 (0.685-1.471)  0.577 (0.397-0.839) | 0.021  0.553  0.449  0.668  0.225  0.619  0.355  0.869  0.744  0.572  0.085  0.372  0.984  0.004 | 0.975 (0.896-1.060)  0.949 (0.910-0.990)  0.970 (0.921-1.021)  0.917 (0.812-1.035)  1.174 (0.694-1.985)  1.000 (0.999-1.000)  1.098 (0.950-1.268)  0.999 (0.999-1.000)  0.945 (0.861-1.038)  0.921 (0.585-1.449)  Reference  0.881 (0.436-1.780)  1.055 (0.613-1.817)  1.275 (0.775-2.096)  0.699 (0.428-1.141) | 0.547  0.014  0.245  0.160  0.550  0.856  0.205  0.538  0.238  0.721  0.725  0.847  0.339  0.152 | 0.900 (0.744-1.088)  1.137 (0.957-1.352)  1.698 (0.424-6.803)  1.061 (0.911-1.236)  0.542 (0.125-2.347)  1.001 (0.999-1.002)  1.391 (0.977-1.981)  1.001 (1.000-1.002)  0.805 (0.584-1.109)  0.769 (0.232-2.544)  Reference  1.424 (0.135-15.031)  0.754 (0.187-3.033)  1.742 (0.558-5.442)  1.190 (0.365-3.883) | 0.275  0.145  0.454  0.445  0.413  0.103  0.067  0.001  0.185  0.667  0.769  0.691  0.340  0.773 |

OR, odds ratio; CI, confidence interval; BMI, body mass index; AFC, antral follicle count; AMH, anti-mullerian hormone; RSA, recurrent spontaneous abortion; PPOS, progestin-primed ovarian stimulation.
